# *HOXA4* Gene Promoter Hypermethylation as an Epigenetic Mechanism Mediating Resistance to Imatinib Mesylate in Chronic Myeloid Leukemia Patients

**DOI:** 10.1155/2013/129715

**Published:** 2012-12-26

**Authors:** Marjanu Hikmah Elias, Abdul Aziz Baba, Azlan Husin, Sarina Sulong, Rosline Hassan, Goh Ai Sim, S. Fadilah Abdul Wahid, Ravindran Ankathil

**Affiliations:** ^1^Human Genome Centre, School of Medical Sciences, Universiti Sains Malaysia, Health Campus 16150 Kubang Kerian, Kelantan, Malaysia; ^2^Haemato-Oncology Unit, Department of Internal Medicine, School of Medical Sciences, Universiti Sains Malaysia, Health Campus 16150 Kubang Kerian, Kelantan, Malaysia; ^3^Hematology Department, School of Medical Sciences, Universiti Sains Malaysia, Health Campus 16150 Kubang Kerian, Kelantan, Malaysia; ^4^Hospital Pulau Pinang, Malaysia; ^5^Medicine Department & Cell Therapy Centre, UKM Medical Centre, Malaysia

## Abstract

Development of resistance to imatinib mesylate (IM) in chronic myeloid leukemia (CML) patients has emerged as a significant clinical problem. The observation that increased epigenetic silencing of potential tumor suppressor genes correlates with disease progression in some CML patients treated with IM suggests a relationship between epigenetic silencing and resistance development. We hypothesize that promoter hypermethylation of *HOXA4* could be an epigenetic mechanism mediating IM resistance in CML patients. Thus a study was undertaken to investigate the promoter hypermethylation status of *HOXA4* in CML patients on IM treatment and to determine its role in mediating resistance to IM. Genomic DNA was extracted from peripheral blood samples of 95 CML patients (38 good responders and 57 resistant) and 12 normal controls. All samples were bisulfite treated and analysed by methylation-specific high-resolution melt analysis. Compared to the good responders, the *HOXA4* hypermethylation level was significantly higher (*P* = 0.002) in IM-resistant CML patients. On comparing the risk, *HOXA4* hypermethylation was associated with a higher risk for IM resistance (OR 4.658; 95% CI, 1.673–12.971; *P* = 0.003). Thus, it is reasonable to suggest that promoter hypermethylation of *HOXA4* gene could be an epigenetic mechanism mediating IM resistance in CML patients.

## 1. Introduction

Chronic myeloid leukemia (CML) is a myeloproliferative disorder that comprises 14% of all leukemias. The molecular pathogenesis of CML involves the clonal expansion of pluripotent haematopoietic stem cells containing the *BCR-ABL* fusion oncogene. *BCR-ABL* gene results from a reciprocal translocation between chromosome 9 and 22 to form the Philadelphia chromosome [1]. This *BCR-ABL* fusion gene codes for a p210 kD protein with increased tyrosine kinase activity. Imatinib mesylate (IM) or Glivec (NOVARTIS Pharma) is a selective molecular inhibitor of the BCR-ABL oncogene protein and permits long term disease control in about two thirds of chronic phase CML patients [2]. IM has dramatically improved the treatment of CML and is generally considered as frontline therapy for CML patients. Despite its striking efficacy, development of resistance in significant proportion of CML patients on IM therapy has emerged as a major clinical problem affecting both patients and treating physicians. 

Various mechanisms of resistance and suboptimal response to IM have been described, involving *BCR-ABL1*-dependent and *BCR-ABL1*-independent pathways [3, 4]. *BCR-ABL1*-dependent mechanism usually involves point mutations in the tyrosine kinase domain (TKD) and amplification of *BCR-ABL* gene, with mutations in the *BCR-ABL* tyrosine kinase domain being better characterized [5]. Our previous study on *BCR-ABL* TKD mutation analysis showed that *BCR-ABL* mutations accounted for IM resistance in only 21.7% of Malaysian CML patients on IM therapy (communicated separately; in Press). This indicated that *BCR-ABL* mutations are not the only cause for relapse and resistance. It is presumed that the mechanisms of IM resistance in CML patients who do not have TKD mutation might be mediated through *BCR-ABL*-independent pathways. However, the exact mechanism in *BCR-ABL*-independent pathway still remains unclear, despite several genetic and epigenetic mechanisms postulated to be involved in the *BCR-ABL-*independent pathway. 

It is largely known that DNA in cancer cells is very unstable. Epigenetic silencing is a phenomenon whereby gene transcript maybe suppressed through DNA methylation. Gene expression can be strongly modified through epigenetic alteration such as DNA hypo or hypermethylation. DNA methylation at cytosine residues in gene promoter CpG sequences is known to inhibit gene transcription, resulting in decreased protein expression. Genomic instability and DNA modifications certainly confer to the cancer cells, a higher capability of becoming resistant [6]. The human Homeobox (*HOX*) gene network encodes master regulators in haematopoiesis and DNA methylation has been implicated to have an important role in aberrant control of *HOX* gene expression [7]. Inappropriate expression of *HOX* gene has been implicated in development of hematopoietic malignancies. Methylation of a *HOX* gene, *HOXA4* has been strongly associated with progression to blast crisis and poor response to treatment in other types of leukemia patients [7]. 

In CML, increased epigenetic silencing of potential tumor suppressor genes has been found to be correlated with disease progression in a small proportion of patients treated with Imatinib [8]. This suggests the possibility of a relationship between epigenetic silencing and development of IM resistance. Few studies have suggested that hypermethylation might play a role in disease progression in CML. It could be plausible that changes in gene silencing by DNA methylation might play a role in developing alternative routes for cells to circumvent the effects of IM. We hypothesized that promoter hypermethylation of *HOXA4 *could be an epigenetic mechanism which mediate resistance to IM in CML patients. This study was designed to test this hypothesis. 

## 2. Methodology

### 2.1. Patient Samples and Control

The study was undertaken at Hospital Universiti Sains Malaysia, after getting approval from the Research and Ethics Committee of University Sains Malaysia and Ministry of Health (MOH), Malaysia (NMRR-10-1206-7127). A total of 95 Malaysian CML patients during their treatment with IM were enrolled. The patients selected were Philadelphia chromosome positive CML patients in chronic, accelerated, or blast phase, treated for at least 12 months, with IM (400 mg and 600 mg, resp.) on frontline treatment. These CML patients were categorized into IM resistant and IM good responders based on their molecular and/or cytogenetic response. IM-resistant patients were defined as those CML patients showing less than complete cytogenetic response by 12 months and/or lack of attainment of major molecular response by 18 months after initiation of therapy. Secondary resistance was defined as loss of complete cytogenetic response and/or loss of major molecular response.

Three millilitres of peripheral blood from each patient was collected in EDTA tube. Additionally, blood samples from 12 normal healthy controls were also collected and included for analysis. Universal methylated DNA and unmethylated DNA (ZYMO research, USA) were used as 100% and 0% methylation DNA control, respectively. Both types of the DNA were modified with bisulfite treatment and was subsequently mixed according to the ratio of 10%, 25%, 50%, and 75%. This serial methylation percentage was included in each experimental run. 

### 2.2. Genomic DNA Extraction and Sodium Bisulfite Treatment

The genomic DNA of all patients and controls was isolated using the GENTRA PUREGENE Blood Kit (Qiagen, Germany) according to the supplier's recommendation. DNA quantity was identified spectrophotometrically by using NanoQuant Infinite M200 (Tecan, Switzerland) and the quality of the DNA was confirmed by agarose gel electrophoresis using 1% agarose gel. 

After extraction of genomic DNA, 500 ng of the DNA was subjected to bisulfite treatment utilizing the EZ DNA Methylation-Gold Kit (ZYMO Research, USA) following manufacturer's recommendation. Besides the patient samples, universal methylated DNA and unmethylated DNA were also treated with bisulfite using the same kit. Before mixing the methylated and unmethylated controls into 10%, 25%, 50%, and 75% percentages, the concentration of bisulfite treated DNA control samples were carefully measured at a value of 40 *μ*g/mL for Ab_260_ = 1.0 (the wavelength used corresponds to RNA wavelength as the recovered bisulfite-treated DNA was single stranded with limited non-specific base-pairing at room temperature) (ZYMO research, USA). The concentration of the eluted bisulfite treated DNA samples of all patients was also measured and the final concentration used was 20 ng for MS-HRM analysis.

### 2.3. Primer Design

**Figure 1:**
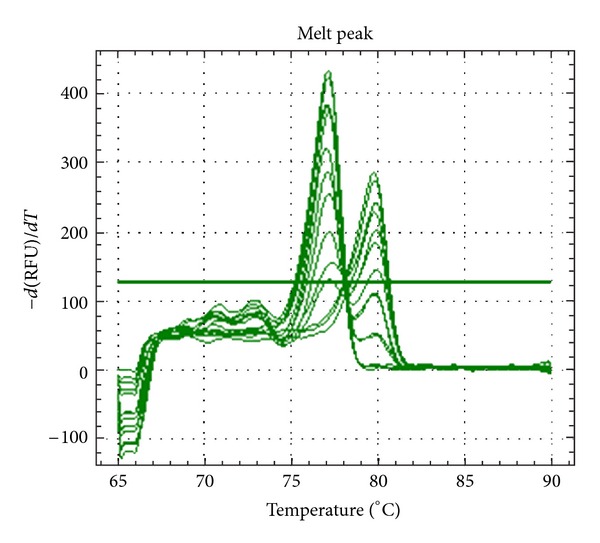
Derivative Melt peak of the serial percentage of methylation control produced two specific peaks which represent the unmethylated (approximately 77°C) and methylated (approximately 80°C) PCR product. Fully unmethylated sample produced only unmethylated peak, 100% methylated sample produced only methylated peak and samples with mixture of unmethylated, and methylated displayed both peaks.

Primers were designed based on criteria stated by Wojdacz et al. [9] with some additional modifications using the Methyl Primer Express v1.0 Software (Applied Biosystem, USA). While designing the primers, the following points were considered. The primers should amplify 100 to 150 bp PCR product with only one CpG dinucleotides each, as more CpG dinucleotide in the primer sequence was found to promote bias amplification towards the methylated template. The primers also should amplify both methylated and unmethylated sequence simultaneously (Figure 1). The CpG in the primers should not be at 3′-end, and preferably, it should be placed as close as possible to the 5′-end of the primer. However, CpG situated in the middle (at least 5th nucleotide from the 3′-end) could also be acceptable provided it could produce good melt curve differentiation [10]. To ensure that the bisulfite converted DNA specific amplification and to prevent amplification of unconverted DNA template, the 3′-end of primers should contain one or more Ts derived from the non-CpG after bisulfite treatment. Accordingly the primers sequences designed for our study was 5′-TTTTGAAGGATA$\underline{\underline{C}}$GAAGTTTGA-3′ (forward primer) and 5′-TCCTCTC$\underline{\underline{G}}$ AAAACCCTCTAC-3′ (reverse primer) for *HOXA4 *promoter.

### 2.4. Validation of Designed Primer

Subsequent to primer design, the forward and reverse primer sequences were tested for their possible secondary structure, self dimer and hetero dimer formation using OligoAnalyzer 3.1 Software (http://eu.idtdna.com/analyzer/Applications/OligoAnalyzer/Default.aspx). As Δ*G* less than −7 may form a very stable primer dimer, primer sequence with Δ*G* higher than −7 was chosen. The higher the Δ*G* (more than −3.5) the better it seemed, as it could subordinate the primer dimer problem. 

The computational prediction of the melting curve as well as the derivative melting curve shape was also derived on the sequence of the PCR product generated, using algorithm like the uMelt v2.0.2 (http://www.dna.utah.edu/umelt/um.php). By using this algorithm, the expected melting temperature of the PCR product was of help in forecasting the melting curve temperature adjustment during the optimization of the laboratory work. Care was taken to see that the derivative melting peak also had only one specific peak without any shoulder at the adjacent slope. PCR amplicon with several melting peaks would be showing the presence of multiple melting domains and may produce complex melting profile that maybe hard to interpret. 

A sequence similarity search program designed to explore in silico bisulfite modified DNA (either methylated or not at its CpG dinucleotides) was used to confirm the amplification specificity of the designed primer. The primers were blast before synthesised, using the methBLAST software (http://medgen.ugent.be/methBLAST/). 

### 2.5. High-Resolution Melt Analysis

**Figure 2:**
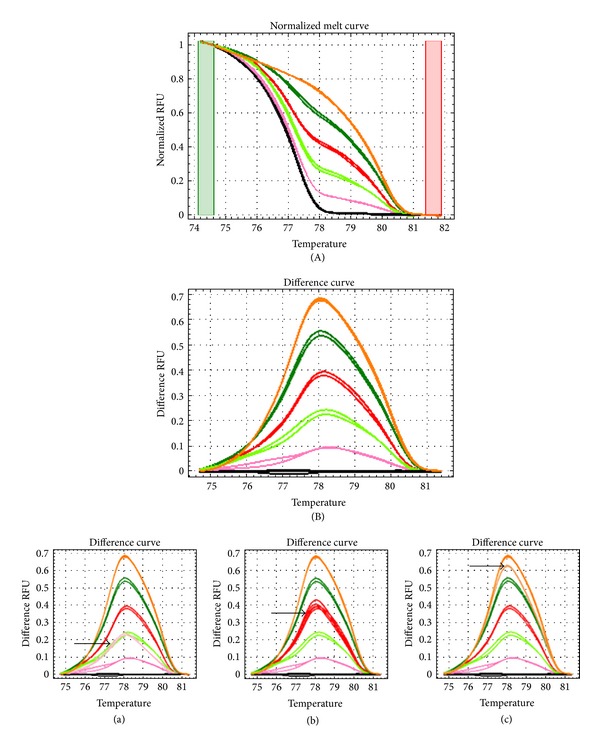
HRM curves for HOXA4 methylation standard. (A) Normalized melt curve of HOXA4 methylation standards in the form of serial methylation percentage (0% black lines, 10% pink line, 25% green line, 50% red line, 75% dark green line, and 100% orange line. (B) Melting curves were normalized to the 0% methylation standards and the standard melt curve was used as the marker for identifying the methylation percentage of samples. For example: (a) ~25% methylated, (b) ~50% methylated, and (c) 75%–100% methylated samples.

PCR amplification and MS-HRM analysis were performed using CFX Real Time PCR Detection System (Bio-Rad Laboratories, USA). The PCR amplifications were performed and monitored using the CFX Manager Software and the HRM data was analysed with the Bio-Rad Precision Melt Analysis. PCR amplification was performed in a total volume of 10 *μ*L, containing 1x Precision Melt Supermix (Bio-Rad Laboratories, USA), 200 nM of each designed primer, and 20 ng of bisulfite treated DNA template. All samples and DNA percentage controls were performed in triplicate. The PCR condition was started at 95°C for 2 minutes for initial denaturation, followed by 50 cycles of 10 seconds at 95°C for denaturation, 30 seconds at 50°C for annealing and another 30 seconds at 72°C for extension. The PCR amplification was then followed by heteroduplex formation at 95°C for 30 seconds and subsequently 60°C for 1 minute. The high-resolution melting analysis was performed immediately afterwards by increasing the temperature from 65°C to 95°C for 10 seconds at each step with the 0.2°C increments. For each run, a no template control (NTC) and serial percentage control (0%, 10%, 25%, 50%, 75%, and 100%) in triplicate were included (Figure 2).

The annealing temperature during the PCR amplification was gradiently optimized as it could create amplification bias in MS-HRM. Higher annealing temperature could introduce bias towards the amplification of methylated template. The most preferable annealing temperature would be the one that could differentiate between the serial percentage controls. Hence, multiple annealing temperatures with mixtures of methylated controls were tested and the best fit standard melting curve was selected.

### 2.6. Statistical Analysis

Unconditional logistic regression analysis was used to assess the relationship between *HOXA4* promoter methylation percentage and the response of CML patients to IM by calculating the Odd Ratios (ORs) and 95% Confidence Interval (CI). The test was conducted by SPSS software with all *P* values as two-sided.

## 3. Results

A total of 95 samples including both IM resistant (*n* = 57) and IM good response (*n* = 38) CML patients and 12 samples from normal control donors were tested for methylation percentage employing the methylation-specific high-resolution melt analysis (MS-HRM analysis). All IM-resistant CML patients were initially screened for *BCR-ABL* TKD mutations and those who showed mutations were excluded from MS-HRM analysis. Fifty seven (57) IM-resistant CML patients without *BCR-ABL* mutations were subjected to *HOXA4* methylation analysis. For comparison, 38 CML patients showing good response to IM and 12 normal controls were also subjected to *HOXA4* methylation analysis. Thus, in this report, IM-resistant CML patients are relatively higher than good response CML patients (57 versus 38). Out of 57 IM-resistant CML patients, 22 were males and 35 were females with mean age of 45 years. In the case of 38 IM good response CML patients, 20 were males and 18 were females with mean age of 36 years. Among the IM-resistant CML patients, 48 patients were categorized into primary resistance group and 9 patients were categorized into secondary resistance group. 

Methylation percentage of the promoter region of *HOXA4 *gene in the normal controls was in the range of 10% to 49%. In the case of whole group of 95 CML patients, the *HOXA4* promoter methylation was in the range of 10% to 100% with most of them showing dense range of more than 50% methylation.  Table 1 shows the methylation percentage frequencies of *HOXA4* gene promoter in normal controls and CML patients, in which the methylation percentages were subdivided into four categories. Except for the low level category (0–24%), the percentages of methylation levels in other 3 categories were significantly higher in CML cases in comparison to control.

When the *HOXA4* methylation profile among CML patients showing good response and resistance to IM was evaluated separately, hypermethylation was found to be significantly less dense in IM good response CML patients, compared to IM-resistant CML patients. However, when the methylation percentages of *HOXA4 *were categorized into two classes, 1–49% as methylated and 50–100% as hypermethylated and the values were compared, *HOXA4* hypermethylation was significantly higher among IM-resistant CML patients (*P* = 0.002) than IM good response CML patients. Furthermore, when the risk association of the two methylation categories (methylated and hypermethylated) with IM resistance was evaluated, *HOXA4* hypermethylation was found to be associated with a significantly higher risk for IM resistance with OR, 4.658 (95% CI, 1.673–12.971; *P* = 0.003) as shown in Table 2.

## 4. Discussion

DNA promoter hypermethylation is a powerful mechanism of tumor-suppressor gene silencing that mediates neoplastic transformation [11]. Despite CML starts as a genetically homogeneous disease, it has been hypothesized that disease progression and clinical heterogeneity in CML are related to epigenetic factors including DNA hypermethylation. Hypermethylation in several tumor-suppressor genes (i.e., *TFAP2A* and *EBF2*) had been reported in CML patients on disease progression [12]. Recently, Jelinek et al., [8] observed a higher frequency of hypermethylation in *OSCP1* and *NPM2* genes among CML patients who were resistant or intolerant to IM. However, there are still no reports available on the involvement of *HOX* gene family hypermethylation in mediating resistance to IM.

The *HOX* gene family consisting of 39 genes are a large family of homeodomain containing transcription factors which regulate developmental process, haematopoietic differentiation, and leukemogenesis. *HOX* gene translocations are observed frequently in leukemia. Majority of the *HOX* genes have CpG islands at their transcription start site (TSS) regions. Silencing of *HOX* genes by DNA methylation are thought to disrupt normal development of blood cells and thus to be involved in leukemic transformation [13]. Hence, compared to other protooncogenes, hypermethylation of* HOX* genes might affect the CML transformation.

By utilizing MS-HRM, the *HOXA4* promoter methylation quantification showed a distribution profile of 10% to 100% methylation, with none of the samples showing 0% methylation. Samples from normal individuals showed methylation of 10% to 49% whereas samples from CML patients showed methylation of 10% up to 100%. 

However, based on our experience as well as from the literature, designing of the primer was found to be the most crucial part in ensuring that the methylation percentage be clearly differentiated [10]. Our experience prompt us to suggest that, in order to amplify *HOXA4* promoter for methylation profiling, the methylation-specific primers should have only one CpG site in the forward and reverse primer, respectively. By considering this kind of factor in primer designing, methylation percentage ranging from 0% to 75% could be clearly differentiated. However, samples that show 75%–100% hypermethylation cannot be clearly differentiated among themselves. 

In leukemia-free normal population, few researchers showed absence of methylation, whereas few other studies showed a very low/absence of methylation [14]. However, in the present study, normal samples showed a range of 10% to 49% methylation level at the promoter region of *HOXA4*. Because of this, samples that showed methylation level of 50%–100% only were considered as *HOXA4 *hypermethylated samples. 

There are reports correlating hypermethylation of* HOXA4* with the development of leukemia. Zangenberg et al. [15] reported that 77% of their acute myeloid leukemia (AML) patients exhibited hypermethylation of *HOXA4 *promoter region. Apart from AML, another study had demonstrated the contribution of *HOXA4* promoter hypermethylation in chronic lymphoid leukemia (CLL) [7]. Furthermore,* HOXA4* hypermethylation has been demonstrated to be usually associated with the progression of CML to blast phase and play an important role in the development of leukemia [7]. 

In the current study, we further evaluated whether *HOXA4* hypermethylation induced gene silencing could be an alternative mechanism of CML cells to circumvent the effects of IM and thereby develop resistance to IM. To the best of our knowledge, no previous reports are available on the involvement of *HOXA4* in mediating IM resistance among CML patients, ours being the first of its kind. Interestingly, in our study, *HOXA4* hypermethylation level of 50–100% was significantly higher (*P* = 0.002) among IM-resistant CML patients compared to IM good response CML patients. When the association between *HOXA4* hypermethylation and IM resistance was examined, patients with *HOXA4* promoter hypermethylation level in 50–100% showed a significantly higher risk for IM resistance (OR = 4.658; 95% CI, 1.673–12.971; *P* value, 0.003). Thus, hypermethylation of *HOXA4* may be a marker of resistance to IM. However, mechanistic studies are still needed to confirm if hypermethylation of *HOXA4* is indeed causing poor response to IM.

The treatment mechanism of IM involves the arrest of *BCR-ABL* dynamic activity. No known mechanism of IM activity towards *HOXA4* has been literally reported so far. Hypermethylation of *HOXA4* has been found to promote inactivation of gene expression [15, 16]. As HOXA4 protein is a DNA-binding transcription factor which may regulate gene expression, morphogenesis, and differentiation, it is reasonable to suggest that the suppression of HOXA4 protein production by hypermethylation induced gene silencing could be one of the potential mechanisms in *BCR-ABL*-independent pathway that promote IM resistance in CML patients. Moreover, Fournier et al. demonstrated the potential of *HOXA4* retrovirus-mediated over expression of haematopoietic stem cell to give rise to mature myeloid progeny [17]. In ovarian cancer, several studies reported that *HOXA4* plays an important role in cell motility, spreading and cell-cell adhesion [18, 19]. Based on those reports, it is reasonable to suggest that suppression of HOXA4 protein might be impairing the normal development as well as proliferation of myeloid progeny and could be a potential epigenetic mechanism in BCR-ABL-independent pathway in promoting IM resistance among CML patients. 

It is reasonable to suggest that hypermethylation of *HOXA4* gene might be circumventing the clinical response to IM and thus playing an important role as inhibitor to normal leukemogenesis. This data contributes to a new understanding of epigenetic mechanism also as a mediator in resistance development to IM in CML patients. Inhibition of this process may have potential as better therapy and warrants the need of utilizing hypomethylating agents for CML patients showing this epigenetic mechanism of resistance. Thus, hypermethylation profile of *HOXA4* gene also could be considered as an epigenetic biomarker, in addition to the *BCR-ABL* gene mutations analysis, for prediction of response to IM treatment among CML patients.

## Figures and Tables

**Table 1:** Methylation percentage frequencies of *HOXA4* gene in IM resistant and good response CML patients.

*HOXA4* methylation (%)	Normal Control	CML Patients	*P* value	CML Patients		
				Good Response	Resistance	*P* value
0–24	6	6	0.000*	4	2	0.213
25–49	6	16	0.016*	11	5	0.010*
50–74	0	43	0.001*	18	25	0.736
75–100	0	30	0.018*	5	25	0.002*

*Chi-Square test, *P* < 0.05 significant at 95% CI.

**Table 2:** Risk association between *HOXA4* methylation status and IM response among CML patients.

*HOXA4 * methylation (%)	CML Patients on IM therapy			*P* value	OR (95% CI)
	Good Response	Resistance	Patients Total
0–49	15	7	22	—	Reference
50–100	23	50	73	0.003*	4.658 (1.673–12.971)

*Chi-Square test, *P* < 0.05 significant at 95% CI.
